# An Automatic Estimation of Arterial Input Function Based on Multi-Stream 3D CNN

**DOI:** 10.3389/fninf.2019.00049

**Published:** 2019-07-05

**Authors:** Shengyu Fan, Yueyan Bian, Erling Wang, Yan Kang, Danny J. J. Wang, Qi Yang, Xunming Ji

**Affiliations:** ^1^School of Sino-Dutch Biomedical and Information Engineering, Northeastern University, Shenyang, China; ^2^Neusoft Institute of Intelligent Medical Research, Shenyang, China; ^3^Engineering Research Center for Medical Imaging and Intelligent Analysis, National Education Ministry, Shenyang, China; ^4^Department of Radiology, Xuanwu Hospital, Capital Medical University, Beijing, China; ^5^Laboratory of FMRI Technology, Keck School of Medicine, Mark and Mary Stevens Neuroimaging and Informatics Institute, University of Southern California, Los Angeles, CA, United States

**Keywords:** AIF, multi-stream, 3D CNN, perfusion, MRI

## Abstract

Arterial input function (AIF) is estimated from perfusion images as a basic curve for the following deconvolution process to calculate hemodynamic variables to evaluate vascular status of tissues. However, estimation of AIF is currently based on manual annotations with prior knowledge. We propose an automatic estimation of AIF in perfusion images based on a multi-stream 3D CNN, which combined spatial and temporal features together to estimate the AIF ROI. The model is trained by manual annotations. The proposed method was trained and tested with 100 cases of perfusion-weighted imaging. The result was evaluated by dice similarity coefficient, which reached 0.79. The trained model had a better performance than the traditional method. After segmentation of the AIF ROI, the AIF was calculated by the average of all voxels in the ROI. We compared the AIF result with the manual and traditional methods, and the parameters of further processing of AIF, such as time to the maximum of the tissue residue function (Tmax), relative cerebral blood flow, and mismatch volume, which are calculated in the Section Results. The result had a better performance, the average mismatch volume reached 93.32% of the manual method, while the other methods reached 85.04 and 83.04%. We have applied the method on the cloud platform, Estroke, and the local version of its software, NeuBrainCare, which can evaluate the volume of the ischemic penumbra, the volume of the infarct core, and the ratio of mismatch between perfusion and diffusion images to help make treatment decisions, when the mismatch ratio is abnormal.

## Introduction

In recent years, ischemic stroke has become a tremendous health problem all over the world (Naghavi et al., 2017). Stroke incidence in China has increased yearly and stroke has become the leading cause of death (Li et al., 2015; Zhou et al., 2016). The key to the treatment of stroke is to rescue the ischemic penumbra using advanced imaging techniques, such as CT/MR perfusion imaging (Hakim, 1998). However, physicians in suburban hospitals cannot accurately identify the ischemic penumbra due to the lack of experience in imaging interpretation, leading to significant delays in stroke treatment. Hence, enhancing the capabilities of physicians capabilities coming from these hospitals is of great significance (Bjørnerud and Emblem, 2010). In this study, we aimed to setup a platform based on novel arterial input function (AIF) methodology on perfusion CT/MRI which enables automatic ischemic penumbra evaluation.

Perfusion-weighted imaging (PWI) can be used to assess perfusion parameters for noninvasive diagnosis of stroke conditions. This method involves monitoring the continuous changes of the time density curves (TDCs) of a bolus tracer passing though the capillary bed over time. Quantitative analysis using dynamic susceptibility contrast (DSC) MRI perfusion requires determination of the AIF, which is the concentration of the contrast agent over time in a brain-feeding artery. The tissue TDC can be considered as a convolution of the response function with the AIF. To analyze ischemic tissue, the response function which can be calculated by deconvolution with the AIF is necessary. We operated a deconvolution with the TDC on each voxel to obtain hemodynamic maps containing cerebral blood flow (CBF), cerebral blood volume (CBV), time to maximum of the tissue residue function (Tmax), and mean transit time (MTT). The characteristic TDC of a voxel in a major arterial vessel (like the Basal Artery or the Internal Carotid Artery) is considered as AIF, which is known as a reference curve to calculate hemodynamic maps. The AIF is a key reference curve used in the deconvolution model to obtain quantitative CBF, CBV, Tmax, and MTT estimation. As it is the reference curve, AIF has a great influence on the result of the deconvolution operation. To improve reliability, quality, and reproducibility of the AIF estimation, several approaches have been proposed, including alternative measurement techniques such as application of imaging protocols or data processing. Lorenz and Calamante proposed a local AIF extraction method to replace the global AIF (Grüner et al., 2006; Lorenz et al., 2006; Willats et al., 2011). R. Gruner used the theory of homomorphic transformations and complex cepstrum analysis to obtain a voxel-specific AIF (Lorenz, 2004). Murase estimated the AIF using fuzzy clustering for quantification of CBF (Calamante et al., 2004). Chen incorporated knowledge about artery structure, fluid kinetics, and the dynamic temporal property to find the AIF (Zhu et al., 2011). Peruzzo et al. (2011) draws a ROI, then uses a recursive cluster analysis on the ROI to select the arterial voxels. From all these previous studies we realized that deep learning has not yet been used for AIF extraction, and therefore we proposed a network to extract the AIF and compared our method with the traditional method and a combination of Unet3D and fuzzy c-means.

The AIF obtained from a single voxel or a small region is not reliable enough, since noise in spatial measurements and motion in temporal measurements affect the AIF estimation. Therefore, it is more appropriate to extract the AIF in a region or volume (Bleeker et al., 2011; Shi et al., 2014). In addition, the spatial resolution of perfusion sequences is low, making it difficult to identify vessels. Therefore, the selection of AIF depends on the expertise, experience, and skill of experts. High time consumption and low reproducibility are the biggest disadvantages of manual selection of the AIF. Some approaches have been proposed to partially or fully automate AIF estimation (Alsop et al., 2002). Murase et al. (2001), Van Osch et al. (2001), and Duhamel et al. (2006) extracted the AIF using cluster method, but a ROI should be marked manually prior to AIF extraction. Reishofer et al. (2003) extracted the AIF by classification using criteria which involved inherent features of the arterial input, such as an early bolus arrival and a fast passage, as well as a high contrast agent concentration.

## Materials and Methods

### Manual Arterial Input Function Annotation

For manual AIF annotation, the investigator selects an AIF with the cursor and marks the position of the AIF on the PWI data. In the meanwhile he checks the corresponding concentration curve of the bolus tracer. The investigator first selects a region of interest associated with the main feeding vessel, such as the middle cerebral artery. The TDC is displayed according to the pixel as the cursor moves. The investigator determines the pixel location in the region of interest, when the curve is consistent with the AIF characteristics. Subjectively, the ideal AIF is defined as a curve with large amplitude, small width, fast attenuation, and it can also be described as a gamma variate function fitted to the bolus tracer TDC.

### 3D Convolution

In a 2D network, convolutions only compute features in a plane on the images. It is not applicable to perfusion data analysis, which must extract features in multiple volumes on spatial dimensions and features in multiple frames on the temporal dimension, since 2D convolution can only compute features on static images. 3D CNN is more efficient for temporal features learning than 2D convolution (Prasoon et al., 2013; Kamnitsas et al., 2015; Pereira et al., 2015).

#### Multi-Stream 3D CNN

Perfusion data are 4D data, both spatial and temporal features play an important role in AIF ROI estimation. 3D convolutions alone cannot perform well in the temporal dimension. Since perfusion data are 4D data, it is difficult to process it in a single network. In order to fuse spatial features with temporal features into our network, we applied a multi-stream 3D CNN that processes information on both dimensions. Thus, our network performs operations on the input volume data in both streams simultaneously. Spatial features such as location information in brain tissue are extracted in the first stream, while temporal features such as the TDC information are captured in the second.

The spatial network operates on spatial volumes with a size of *f* × *s* × *w* × *h*, where *f* denotes the number of frames, *s* denotes the number of slices, and *w* and *h* denote the width and height of a single slice. The static appearance by itself is a useful clue, since some features are strongly associated with arteries. A spatial network is essentially a classification or segmentation architecture. The spatial network consists of eight convolution layers, five max-pooling layers, and two fully connected layers, followed by a softmax output layer. Convolution layers are all using 3D convolution with 3 × 3 × 3 kernels and stride 1 in each dimension. The number of filters in each of the eight convolution layers is 64, 128, 256, 256, 512, 512, 512, and 512, respectively. All max-pooling layers are using 3D pooling with 2 × 2 × 2 kernels, because the cube is treated as a 3D volume. There are 4096 units in both fully connected layers.

The temporal network operates on data frames with a size of *s* × *f* × *w* × *h*, where *s* denotes the number of slices, *f* denotes the number of frames, and *w* and *h* denote the width and height of a single slice. This network is therefore different from the spatial network. The dynamic information is obtained to measure the TDC. The temporal network consists of convolution layers, max-pooling layers, and fully connected layers, with numbers of layers of 8, 5, and 2, respectively; and followed by a softmax output layer. All convolution kernels and strides are the same as in the spatial network. All pooling layers have 2 × 2 × 2 kernels, except the first max-pooling layer with a 2 × 2 × 1 kernel, and stride 2 × 2 × 1, in order to retain the temporal information in the early stage and avoid losing it in the convolution process, since the cube is treated as a frame volume. There are 4096 units in both fully connected layers. Each stream is implemented using a 3D CNN, the softmax function converts a raw value into a posterior probability as a softmax score, and softmax scores of each stream are combined by late fusion. The fusion method we chose is the linear support vector machine (SVM) instead of a full connected layer.

**FIGURE 1:**
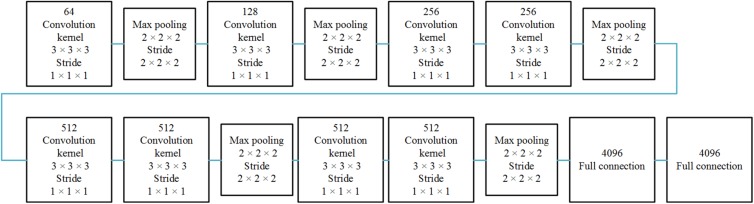
Single 3D CNN network architecture. The 3D CNN network architecture includes eight convolutional layers, five pooling layers, and two full connected layers.

**FIGURE 2:**
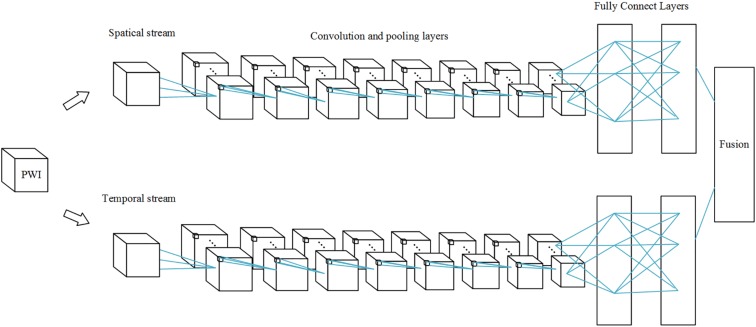
Multi-stream 3D CNN network. Each stream is a 3D CNN network, and the streams are combined by a fusion layer using linear SVM.

The 3D CNN is shown in Figure 1. Since the spatial network and the temporal network are similar, we illustrated only one 3D CNN network. The multi-stream network is shown in Figure 2.

**FIGURE 3:**
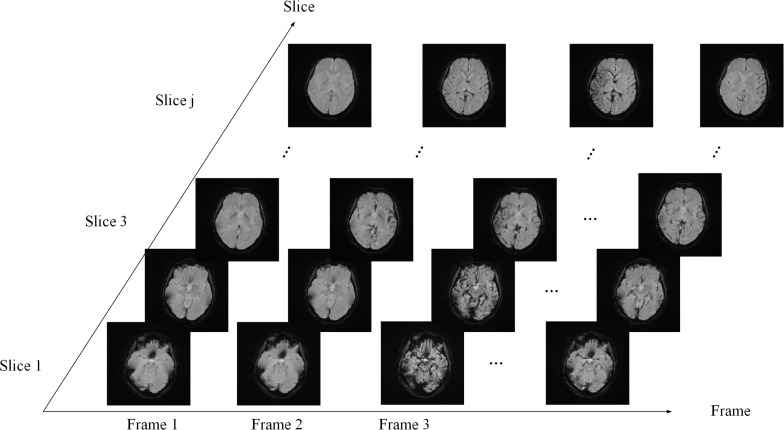
Data sequence order. The sequence is arranged slice by slice in each frame and then arranged frame by frame.

**FIGURE 4:**
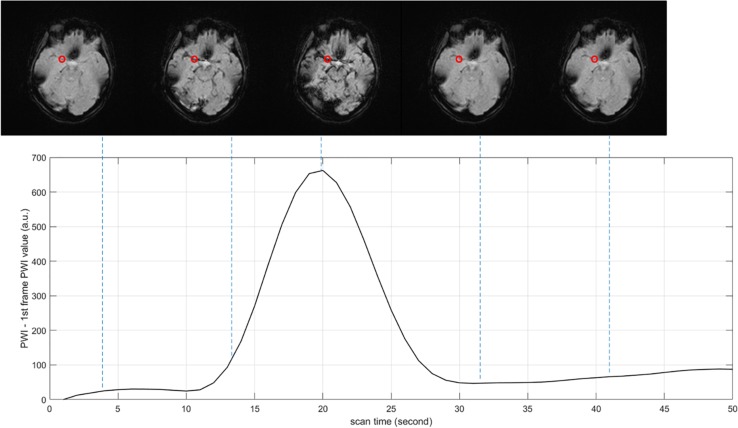
Arterial input function (AIF) curve extracted from time series. The red point shows the location where AIF is extracted, the value of PWI decreases first and then increases with time.

PWI data are arranged in the order of frame volumes, as shown in Figure 3. We rearranged the data in two dimensions, slice and frame. Spatial network input should be arranged frame by frame and slice by slice to locate the ROI. Temporal network input should be arranged slice by slice and then frame by frame, since AIF curves can only be extracted from time series, as shown in Figure 4.

#### Training

The training of the multi-stream 3D CNN framework for the segmentation of the AIF is done in two steps: manual labeling ROI’s perfusion data and auto-labeling based on similarities among the TDCs.

For labeling, we first proceeded to a manual annotation of the ROI which is used to extract the AIF. Thereafter, we calculated the similarity between the TDCs and the AIFs in a neighborhood, then we input the label into the framework. We labeled each volume into two classes of regions, namely AIF vessels and no AIF vessels, respectively.

We constructed an architecture in this paper to segment the AIF vessels in the perfusion volume. The input image is the 3D volume region. To improve the performance of the 3D CNN in this case, we built the multi-stream model with the spatial and temporal networks. Then, the final probability map is fused together. The loss function over all training datasets was minimized through a mini-batch gradient descent approach, and the minimum batch size was 50 inputs. The spatial learning process goes through 50 epochs with a learning rate of 0.001 and a gradient momentum of 0.9. The same parameter settings are used for epoch number, learning rate, and gradient momentum in the temporal learning process.

### Arterial Input Function Extraction

After the segmentation of the AIF vessels, we calculated the AIF by averaging all TDCs of voxels in the classified vessels.

## Experiments

### Data Preparation and Pre-processing

In this study, we collected 100 PWI cases in which 30 were healthy cases and 70 were stroke cases. They were used to train and evaluate the performance of the different methods. Sixty PWI cases among the total dataset were acquired on a 1.5T Discovery MR750 GE MRI scanner with contrast agent at a parameter setting of a TE = 2.6 ms, a TR = 22 ms, and a flip angle = 20-degree. The voxel size is 0.43 × 0.43 × 5.00mm^3^, and each volume contains 512 × 512 × 20 × 50 voxels, corresponding to the width, the height, the number of slices, and the number of frames, respectively. The other 40 PWI cases were acquired on a 3T Verio SIEMENS MRI scanner with contrast agent at a parameter setting of a TE = 3.6 ms, a TR = 21 ms, and a flip angle = 18-degree, their voxel size is 0.43 × 0.43 × 6.50mm^3^ and each volume size is 512 × 512 × 18 × 35.

The preprocessing for perfusion data includes skull removal, motion correction, slice time correction, spatial smoothing, global drift removal, which are general preprocessing stages for perfusion data. To reduce the impact of the brain skull, each dataset was preprocessed to remove the brain skull using the BET2 method (Wels et al., 2009). Motion correction was performed by registering all the volumes in the time series with the multiplicative intrinsic component optimization algorithm (Studholme et al., 1999). We used interpolation to obtain the data of brain slices at the same time point. Spatial smoothing is mainly achieved by low-pass filtering, since many researchers use Gaussian filtering or average filtering, which performances have almost no difference with the BM3D and NLM denoizing results in estimating the AIF.

### Hardware Settings

In this paper, our experiments were implemented, respectively, using MATLAB 2017b and Python 3.0 in Window 10 OS. Environments were made on a desktop computer with eight Intel(R) Xeon(R) CPU E5-1620 v4 @ 3.50 GHz processers, 32 GB of RAM memory, and NVIDIA GeForce GTX 1080.

### Evaluation Method

#### Network Evaluation

Although manual annotations of 100 cases of PWI sequences require a considerable amount of time, in each case we manually segmented the vessels of MIP images on axial planes. The performance of the proposed method in cerebrovascular segmentation is evaluated by comparing MIP post-processed binary images of the proposed method with manual annotations of images on axial planes. Because MIP images on axial planes display most of the blood vessels, the comparison of MIP binary images on axial planes can better illustrate cerebrovascular segmentations differences between the proposed method and manual annotations. Therefore, we evaluated the binary classification performance of our proposed method with parameters such as the accuracy, the sensitivity, the specificity, the precision, and the dice similarity coefficient (DSC), defined as $\mathrm{DSC}=\frac{2\,\left|A\cap B\right|}{\left(\left|A\right|+\left|B\right|\right)}$, where *A* and *B* are ground-truth and segmentations of the AIF, respectively. DSC ranges from 0 to 1.

#### AIF Evaluation

Fuzzy c-means is widely used in determination of the AIF (Jipkate and Gohokar, 2012). Fuzzy c-means clustering was applied to the TDCs, which can be regarded as *n*-dimensional vectors, *n* denoting the frame number of the perfusion data. These vectors were grouped into different clusters. The cluster centroids and the membership matrix were iteratively updated until convergence. A cost function is used to find a cluster closest to the ideal AIF.

Unet3D is a deep learning network applied to 3D data and widely used in biomedical image segmentation. We calculated the maximal intensity projection on the temporal dimension. Then, we segmented the blood vessels by Unet3D and applied fuzzy c-means to determine the AIF only in blood vessels segmented by Unet3D.

Since there is no standard dataset with labeled AIF ROIs, we can only compare all methods with the manual method. The result of the manual method is considered as ground-truth. However, this comparison is not convincing enough, so we compared the further process result by deconvolution: Tmax and rCBF; and it is more persuasive. Subjectively, a single blind investigator will evaluate the shape and location of the AIF result.

The regions in which Tmax is >6 s are considered ischemic regions. CBF is significantly lower in the ischemic region than in the contralateral healthy region. Perfusion–diffusion mismatch is used to identify penumbra in acute stroke. When the apparent diffusion coefficient (ADC) is less than 620, the corresponding region is defined as an infract core in our application. The ischemic region beyond the infarct core is considered to be the ischemic penumbra. Mismatch ratio is the ratio of ischemic penumbra volume to core infarct volume. The larger the ratio is, the more tissues can be saved by treatment. Since the infarct cores were the same in our case, we compared the volumes of the ischemic penumbra and the mismatch ratio obtained by the AIF results, which are presented in our method and the other methods mentioned above.

## Results

We show an example of MIP images on temporal dimensions first and then on spatial dimensions.

The AIFs were estimated by different methods: manual, MS3DCNN, fuzzy c-means, and U-Net3D + fuzzy c-means. Subjectively, a single blinded investigator, who is a Doctor of Medicine working in the Department of Radiology in Xuanwu Hospital of Capital Medical University, which has a high level of neurosurgery and neurology, evaluated the results. Objectively, we compared the AIF location, the curve characteristics of AIF, the perfusion maps, and the mismatch volume estimated by all the other three methods with the same corresponding parameters but estimated by the manual method, respectively.

### The AIF Location

**FIGURE 5:**
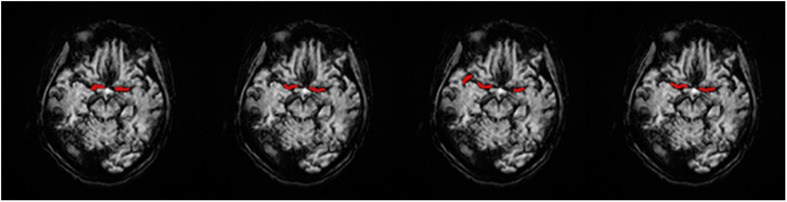
AIF ROI on each AIF masked on MIP in a single slice. **(A)** Manual, **(B)** MS3DCNN, **(C)** Fuzzy c-means, and **(D)** U-Net3D + fuzzy c-means, from left to right.

We obtained each AIF ROI on the maximum density projection, and we compared them, as shown in Figure 5.

Subjectively, the AIF extraction location of all samples calculated by our method in this paper is similar to that of the manual annotation. And since the manual annotation of each doctor will be different, hence the investigator believes that the ROI results of MS3DCNN can be consistent with those obtained from manual annotation.

Objectively, we compared the manual annotated AIF location with those from the three other methods. DSC values were estimated by comparing cerebrovascular segmentations of the MIP images in axial planes with the corresponding manual ground-truths. Values in each column are the average of the testing dataset, as shown in Table 1. Our method has the highest DSC, with a value of 0.7966, while the average DSC value of widely used fuzzy c-means method is only 0.6141, which demonstrates that our method is closest to the ROI of manual annotation. The result was highly consistent with the subjective evaluation of the investigator.

**TABLE 1:** Comparison of DSC value between the automated AIF estimation methods.

**Methods**	**Accuracy**	**Sensitivity**	**Specificity**	**Precision**	**DSC**
Fuzzy c-means	0.9947	0.5073	0.9997	0.9472	0.6141±0.155
U-Net3D + fuzzy c-means	0.9983	0.7620	0.9993	0.8405	0.7941±0.048
MS3DCNN	0.9982	0.7967	0.9991	0.7981	0.7966±0.035

### The Curve Characteristics

**FIGURE 6:**
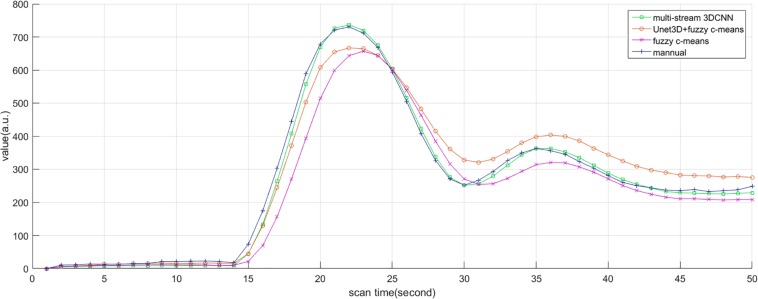
AIF estimated by manual method, multi-stream 3DCNN, Unet3D + fuzzy c-means, and fuzzy c-means. AIF obtained by multi-stream 3D CNN is closest to the manual AIF.

Subjectively, the curve of AIF also conformed to the morphological characteristics mentioned above, such as large amplitude, small width, fast attenuation, and gamma-like shape. In all cases, the investigator believed that AIF obtained by MS3DCNN can have the characteristics mentioned above, as shown in Figure 6. The AIF automatically extracted can be involved in the following perfusion processing by deconvolution.

Due to the different conditions of the patients, including the physical condition, the severity of the disease, the contrast injection time, and the time to start the scan, it is difficult to make statistical analysis of the parameters obtained by direct comparison. So, we only compared the differences of the curve parameters between the AIF extracted by the three automated methods and the AIF extracted by the manual method. The characteristics are amplitude, the center position, and the crest width, so the differences are represented by Δamplitude, Δcenter, and Δwidth. Although we processed all the samples, considering the great number of samples, we only showed 20 of them in Table 2, and calculated the mean and standard deviation for each difference of the parameters, as shown in Table 3. Compared with other methods, MS3DCNN has larger amplitude, higher peak position, fast attenuation, and narrower curve width.

**TABLE 2:** The difference of curve characteristics between the MS3DCNN and the manual method.

**Sample**	**Δamplitude (a.u.)**	**Δcenter (s)**	**Δwidth (s)**
1.	21.51	0.68	0.33
2.	17.08	1.31	0.16
3.	11.83	0.15	0.77
4.	19.74	0.58	0.36
5.	47.47	2.97	1.22
6.	11.38	0.31	0.87
7.	29.05	0.38	1.27
8.	37.21	0.56	0.67
9.	12.82	0.11	0.75
10.	32.60	1.29	0.38
11.	25.21	0.78	0.16
12.	4.31	2.64	2.14
13.	1.23	0.23	0.29
14.	27.29	2.45	0.23
15.	15.25	0.18	0.06
16.	17.82	0.35	1.35
17.	2.70	5.24	2.17
18.	25.51	1.08	0.46
19.	19.26	0.61	0.08
20.	11.77	0.70	0.86
Mean	19.10	1.08	0.71
*SD*	11.04	1.23	0.59

**TABLE 3:** Mean and standard deviation of the difference between the automated methods and the manual method.

**Method**		**Δamplitude (a.u.)**	**Δcenter (s)**	**Δwidth (s)**
MS3DCNN	Mean	18.12	1.05	0.72
	*SD*	11.16	1.21	0.59
Fuzzy c-means	Mean	14.08	1.31	0.90
	*SD*	12.32	1.43	0.75
U-Net3D + fuzzy c-means	Mean	13.98	1.36	0.73
	*SD*	12.21	1.31	0.60

We calculated the similarity of the curves between each automated method and the manual method. The similarity is calculated by Frechet distance, which is greater than or equal to 0. The smaller the Frechet distance between two curves is, the more similar they will be. For better statistical analysis, all the curves should be on the same scale. They were normalized by the peak value of the manual extracted AIF, so the curve value of manual extracted AIF is between 0 and 1, as a reference. The similarity of all samples was calculated and the means and the standard deviation of the similarity were obtained. The mean value of the similarity of our method was lowest, with the value of 0.83, indicating that MS3DCNN method is the closest to the manual method. The standard deviation of our method was also the lowest, with the value of 0.13, indicating that this method is more stable.

### The Perfusion Maps

**FIGURE 7:**
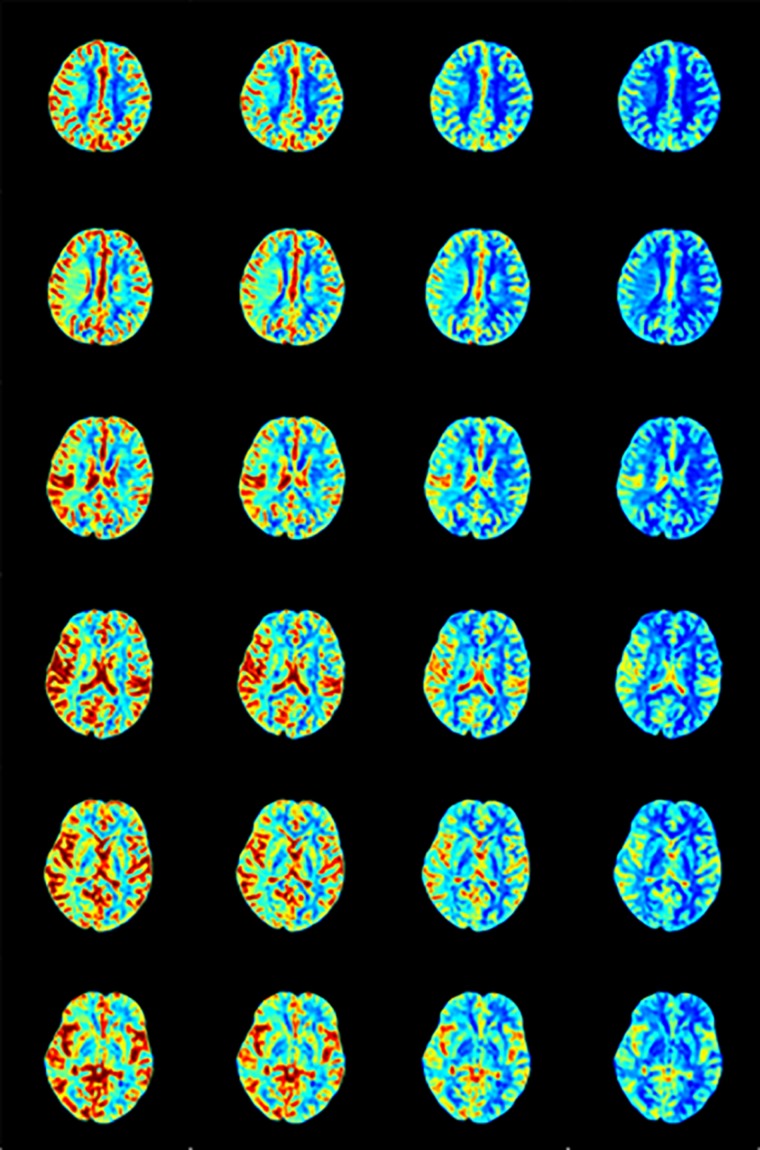
rCBF calculated by AIF from **(A)** Manual, **(B)** MS3DCNN, **(C)** fuzzy c-means, and **(D)** U-Net3D + fuzzy c-means in each column from left to right.

**FIGURE 8:**
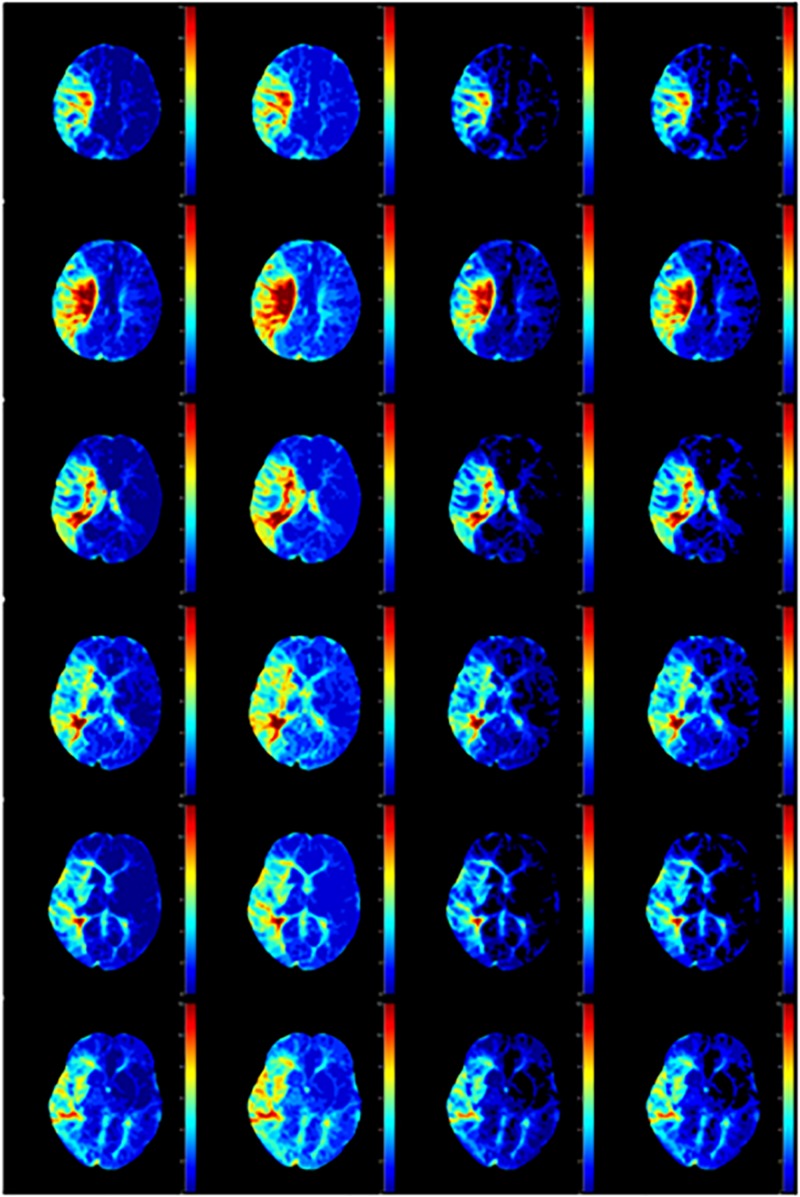
Tmax calculated by AIF from **(A)** Manual, **(B)** MS3DCNN, **(C)** fuzzy c-means, and **(D)** U-Net3D + fuzzy c-means in each column from left to right.

We calculated the response curve to the AIF of each pixel in each sample by deconvolution. Then we calculated the time to peak of response curves as Tmax, and normalized maximum slope as rCBF, collectively known as perfusion maps, as shown in Figures 7, 8.

After observing the perfusion maps and analyzing the patient’s medical history, the investigator concluded that the perfusion maps could be used for diagnosis. The distribution of rCBF and Tmax maps obtained by the same deconvolution processing based on the AIF extracted by our method is basically the same as that of the manual method, the location of ischemic regions can be clearly located through the perfusion maps, while the other two methods have lower Tmax and rCBF, which leads us to underestimate of the size of the ischemic penumbra and the severity of ischemia, respectively.

We still took the perfusion maps calculated by AIF extracted manually as the reference, and made an objective comparison by calculating the difference between the perfusion maps calculated based on AIF extracted by each automatic method and those calculated based on AIF extracted by the manual method. The mean and standard deviation of differences between rCBFs and Tmaxs are shown in Table 4, represented by ΔrCBF andΔTmax.

**TABLE 4:** The difference of Tmax and rCBF between the automated methods and the manual method.

**Method**		**ΔTmax (s)**	**ΔrCBF (a.u.)**
MS3DCNN	Mean	0.79	0.76
	*SD*	0.10	0.11
Fuzzy c-means	Mean	0.43	0.49
	*SD*	0.20	0.28
U-Net3D + fuzzy c-means	Mean	0.39	0.43
	*SD*	0.29	0.24

The Tmax and rCBF values obtained by our method are larger than those obtained by the other two methods, and their performance is consistent with that AIF. The values of Tmax and rCBF are both superior to the latter two methods due to the lower time to peak and the larger peak value of the AIF.

### The Mismatch

We applied our method on a cloud platform, Estroke, http://www.medimagecloud.com/rsplatform/, and the local version of its software, NeuBrainCare, which can calculate the penumbra for stroke perfusion.

**FIGURE 9:**
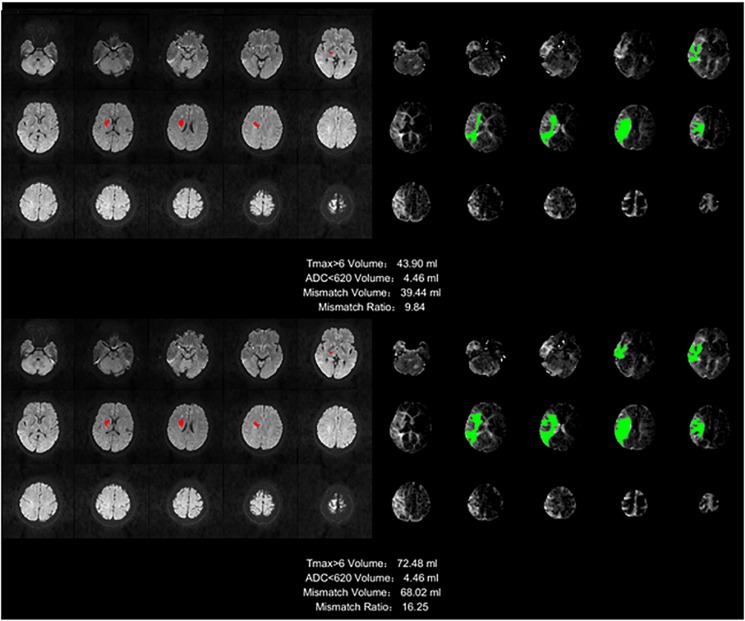
Stroke analysis results of the ischemic region, the infarct core, the mismatch volume, and the mismatch ratio. The infarct core was marked in magenta while the ischemic region was marked in green.

We defined the region with Tmax >6 s as the ischemic region, and the region with ADC which is an additional sequence, less than 620 as the infarct core. The difference between the two volumes was defined as the mismatch volume, and the mismatch volume divided by the ADC < 620 was defined as the mismatch ratio. The larger the mismatch ratio is, the bigger is the volume of brain tissue that can be saved. A mismatch annotation and information was shown in Figure 9, including Tmax > 6 volume (green regions), ADC < 620 volume (red regions), mismatch volume, and mismatch ratio.

The infarct core cannot be found in the image of many samples, so this ratio will be infinite, and for different methods, the ischemic area will be different because of the AIF extracted by different method, but the infarct core based on the ADC is the same. This is the reason we did not compare mismatch ratios, but only compared the mismatch volumes.

The mismatch volume depends on the severity of the stroke. For example, some samples have only small ischemic areas, and others have an entire brain hemisphere tissues with ischemia, there is a huge difference between such samples. For this reason we calculated the mean of the mismatch volumes for all samples with stroke in each method, but without standard deviation, and the ratio to the manual method as the reference was also calculated, as shown in Table 5. The result of our method is the closest to the manual method, with a ratio reaching 93.32%.

**TABLE 5:** The average mismatch volume and ratio to reference of the automated methods and the manual method.

**Method**	**Average mismatch volume (ml)**	**Ratio to reference (%)**
Manual (reference)	48.52	100
MS3DCNN	45.27	93.32
U-Net3D + fuzzy c-means	41.26	85.04
Fuzzy c-means	40.29	83.04

## Discussion

CNN models are deep learning models which have been widely used for object recognition and segmentation. They are usually trained with a large amount of images labeled by humans. But this has not yet been applied for AIF estimation. While automatic AIF estimation only relies on and is heavily influenced by one’s prior knowledge, most of the traditional segmentation methods are unsupervised. These last ones can only extract objects based on observable or expressed features using prior knowledge. In this study, we applied a multi-stream 3D CNN to find the AIF ROI, then we calculate the average curve as AIF.

According to our experimental results, multi-stream 3D CNN has a good performance in AIF ROI segmentation. Different blood vessels share many similar features such as their shapes, while their differences mainly are intensity contrast and vessel thickness. On the timeline, the changes of each voxel are continuous and have large amplitude, small width, fast attenuation, and gamma-like shape. The network extracts features from both the spatial and the temporal parts, followed with a fusion classification to output the result. Moreover, to improve the robustness of the proposed method for different kind of perfusion images, we included from both 1.5T GE and 3.0T SIEMENS, images of healthy and ischemic brain tissue in the training dataset.

Because manual annotations from public datasets were not available, hence we decided to directly compare our method to other network-based segmentation methods. We therefore compare our method to a traditional method, and to a network pre-process method followed by a traditional method. However, in terms of Dice numbers, our unsupervised method shows great potential to perform AIF ROI segmentation.

RAPID is a currently available commercial software which can measure ischemic penumbra. It has been proved effective in several international multicenter clinical trials (Lansberg et al., 2017; Albers et al., 2018; Guenego et al., 2018; Nogueira et al., 2018). The rate of severe disability and death was reduced from 42 to 22% in the thrombectomy group with this advanced imaging software. In our study, our method was applied to Estroke, a cloud-based platform, and the local version of its software, NeuBrainCare, and it could evaluate ischemic penumbra as accurate as RAPID with the datasets from more than 40 hospitals in China. AIF methodology improves the confidence of physicians from suburban hospitals.

## Conclusion

We proposed a new multi-stream 3D CNN network to estimate AIF in brain perfusion images. The model was trained by the labels obtained from manual annotations and similar ROIs based on annotations, which is cost effective in terms of manual efforts. This segmentation framework had a good performance evaluated on perfusion images. The AIF estimation also had a good performance evaluated on PWI data.

## Ethics Statement

Written informed consent was obtained from all participants and all protocols were approved by the Institutional Review Board.

## Author Contributions

SF, YB, and YK designed the model and implementation. EW, XJ, and QY provided the data and was responsible for the analysis and evaluation of the results. DW manually calibrated and designed the experiment. SF wrote the manuscript. YK reviewed the manuscript.

## Conflict of Interest Statement

SF, YB, and YK were employed by Neusoft Institute of Intelligent Healthcare Technology, Co. Ltd. The remaining authors declare that the research was conducted in the absence of any commercial or financial relationships that could be construed as a potential conflict of interest.
